# TDP-43 and FUS mislocalization in VCP mutant motor neurons is reversed by pharmacological inhibition of the VCP D2 ATPase domain

**DOI:** 10.1093/braincomms/fcab166

**Published:** 2021-08-06

**Authors:** Jasmine Harley, Cathleen Hagemann, Andrea Serio, Rickie Patani

**Affiliations:** 1 Department of Neuromuscular Diseases, Queen Square Institute of Neurology, University College London, London WC1N 3BG, UK; 2 The Francis Crick Institute, London NW1 1AT, UK; 3 Centre for Craniofacial & Regenerative Biology, King’s College London, London WC2R 2LS, UK

**Keywords:** amyotrophic lateral sclerosis, motor neurons, RNA binding proteins, valosin-containing protein

## Abstract

RNA binding proteins have been shown to play a key role in the pathogenesis of amyotrophic lateral sclerosis (ALS). Mutations in valosin-containing protein (*VCP/p97*) cause ALS and exhibit the hallmark nuclear-to-cytoplasmic mislocalization of RNA binding proteins (RBPs). However, the mechanism by which mutations in *VCP* lead to this mislocalization of RBPs remains incompletely resolved. To address this, we used human-induced pluripotent stem cell-derived motor neurons carrying *VCP* mutations. We first demonstrate reduced nuclear-to-cytoplasmic ratios of transactive response DNA-binding protein 43 (TDP-43), fused in sarcoma/translocated in liposarcoma (FUS) and splicing factor proline and glutamine rich (SFPQ) in *VCP* mutant motor neurons. Upon closer analysis, we also find these RBPs are mislocalized to motor neuron neurites themselves. To address the hypothesis that altered function of the D2 ATPase domain of VCP causes RBP mislocalization, we used pharmacological inhibition of this domain in control motor neurons and found this does not recapitulate RBP mislocalization phenotypes. However, D2 domain inhibition in *VCP* mutant motor neurons was able to robustly reverse mislocalization of both TDP-43 and FUS, in addition to partially relocalizing SFPQ from the neurites. Together these results argue for a gain-of-function of D2 ATPase in *VCP* mutant human motor neurons driving the mislocalization of TDP-43 and FUS. Our data raise the intriguing possibility of harnessing VCP D2 ATPase inhibitors in the treatment of *VCP*-related ALS.

## Graphical Abstract

**Graphical Abstract:**
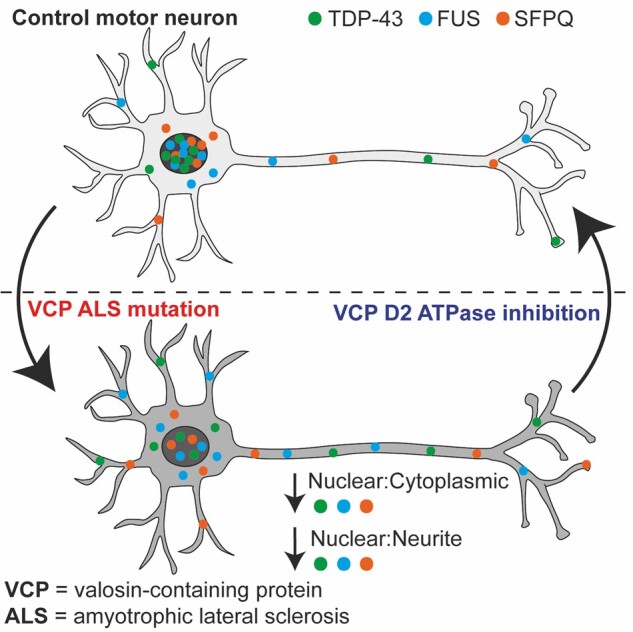

## Teaser

Harley et al. report that mutations in the *VCP* gene that cause amyotrophic lateral sclerosis result in the mislocalization of RNA binding proteins TDP-43, FUS and SFPQ in human motor neurons. Treatment with a VCP D2-ATPase inhibitor reverses the mislocalization of TDP-43 and FUS, suggesting a gain-of-function of D2-ATPase is driving mislocalization.

## Introduction

Amyotrophic lateral sclerosis (ALS) is an invariably fatal neurological disease in which there is selective and progressive degeneration of motor neurons (MNs). Deregulated RNA metabolism, and in particular RNA binding protein (RBP) subcellular localization and function, play a pivotal role in ALS pathogenesis. RBPs orchestrate the RNA life cycle, regulating transcription, splicing, RNA localization, function and decay. Some ALS-causing gene mutations encode RBPs, including transactive response DNA-binding protein 43 (*TARDBP*, which encodes TDP-43), fused in sarcoma/translocated in liposarcoma (*FUS/TLS* or *FUS*) and heterogeneous nuclear ribonucleoprotein A1 (*hnRNPA1*). Subcellular mislocalization of RBPs is also a pathological hallmark of ALS, with TDP-43 mislocalized from the nucleus to the cytoplasm in 97% of ALS cases.^1^ More recently, widespread splicing factor proline and glutamine rich (SFPQ) and FUS mislocalization across different ALS models and sporadic ALS post-mortem tissue has also been reported.^2^^,^^3^ Accumulation of RBPs in the cytoplasm likely contributes to the formation of RBP oligomers and fibrillar pathological cytoplasmic inclusions seen in ALS.^4^^,^^5^ As each RBP can bind to thousands of RNA targets, a disturbance in even one of these proteins potentially has a broad and diverse impact on RNA metabolism.^6^

Valosin-containing protein (VCP or p97) is an abundant AAA+ ATPase (ATPases associated with diverse cellular activities) with a large variety of intracellular functions that encompass almost all aspects of cellular physiology. VCP functions include protein homoeostasis, mitochondrial quality control and apoptosis.^7^ The structure of VCP is important to its many functions; it is a hexameric protein and each subunit has an N-terminal domain, two ATPase domains (D1 and D2) and a disordered C-terminal domain. Autosomal-dominant *VCP* mutations account for 1-2% of familial ALS cases.^8^ Although limited, pathogenic variants in *VCP* have also been identified in sporadic cases of the disease.^9^ Owing to its role in many cellular pathways, disruption to VCP function can lead to several forms of disease. For example, mutations in *VCP* have also been identified in other neurodegenerative diseases, including Inclusion Body Myopathy, Paget’s disease and Frontotemporal Dementia (IBMPFD). Pathogenic mutations of VCP are most commonly found in the N terminal domain, which is responsible for cofactor and ubiquitinated substrate binding and are also present in the D1 and D2 domains.^10^ The majority of VCP mutations have biochemically been shown to associate with a normal or increased ATPase activity in cellular models, with the R155C mutation shown to have more than double the activity of its wildtype counterpart.^11^ However, it remains controversial whether disease mutants with increased ATPase activity cause disease through dominant active or dominant negative mechanisms. Additionally, this has yet to be systematically addressed in patient-derived MNs, which possess the advantage of conveying mutations at pathophysiological levels.

We have previously reported robust differentiation of human-induced pluripotent stem cells (iPSCs) into highly enriched and functionally validated spinal cord MNs in which we have identified time-resolved molecular phenotypes of *VCP*-related ALS.^2^^,^^3^^,^^12^^,^^13^ Here, we used this established human stem cell model of *VCP*-mutation related ALS to first examine the nucleocytoplasmic distribution of key RBPs compared to control MNs. We reveal that TDP-43, FUS and SFPQ exhibit reduced nuclear-to-cytoplasmic ratios in the *VCP* mutant MNs, which extends to an aberrant presence within the neurites. We found that treatment of control MNs with a targeted VCP D2 ATPase inhibitor does not recapitulate ALS RBP mislocalization phenotypes, arguing against a loss of its function in this context. Importantly, we find that in *VCP* mutant MNs the nuclear-to-cytoplasmic mislocalization of both TDP-43 and FUS and the nuclear-to-neurite mislocalization of TDP-43, FUS and SFPQ are reversible by treatment with a pharmacological inhibitor of the VCP D2 ATPase domain. Cumulatively, these findings support a model whereby VCP mutations cause an increase in D2 ATPase activity, which in turn leads to mislocalization of TDP-43, FUS and SFPQ from the nucleus to the cytosol. Our study raises the prospect of harnessing VCP inhibitors that target the D2 ATPase domain in the treatment of VCP-related ALS.

## Materials and methods

### Human fibroblasts and iPSCs

Dermal fibroblasts were cultured in OptiMEM + 10% FCS medium. For iPSC generation, episomal plasmids, pCXLE hOct4 shp53, pCXLE hSK and pCXLE hUL (Addgene) were transfect into dermal fibroblasts. Two control lines used are commercially obtainable (control 2 and control 3) and were purchased from Coriell (cat. Number ND41866*C) and Thermo Fisher Scientific (cat. number A18945). Details of the iPSC lines used in the study can be found in Supplementary Table 1.

### Cell culture and motor neuron differentiation

IPSCs were maintained with Essential 8 Medium media (Life Technologies) on Geltrex (Life Technologies), and passaged using EDTA (Life Technologies, 0.5 mM). IPSC cultures were kept at 37°C and 5% carbon dioxide. IPSCs underwent differentiation into spinal cord MNs as described in Hall et al.^12^ Full details can be found in the Supplementary Information.

### Inhibitor treatment

Motor neuron cultures were treated with 1 μM of ML240 (Sigma; SML1071; CAS: 1346527-98-7) for 2 h.

### Immunofluorescence staining

Cells were fixed in 4% paraformaldehyde in PBS for 15 min at room temperature (RT). For permeabilization and non-specific antibody blocking, 0.3% Triton-X containing 5% bovine serum albumin (BSA) (Sigma) in PBS was added for 60 min. Primary antibodies were made up in 5% BSA and then applied overnight at 4°C. Primary antibodies used were SMI-32 (BioLegend; 801701; mouse; 1:1000), ChAT (Millipore; AB144P; goat; 1:100), βIII-tubulin (abcam; ab41489; chicken; 1:1000), TDP-43 (ProteinTech; 12892-1-AP; rabbit; 1:400), SFPQ (abcam; ab11825; mouse; 1:400), FUS (Santa Cruz; sc-47711; mouse; 1:200), hnRNPA1 (Cell Signaling; 8443S; rabbit; 1:500) and hnRNPK (Santa Cruz; sc-28380; mouse; 1:500). A species-specific Alexa Fluor-conjugated secondary antibody (Life Technologies) at 1:1000 dilution in 5% BSA was added for 90 min at RT in the dark. Cells were washed once in PBS containing DAPI, 4′,6-diamidino-2-phenylindole nuclear stain (1:1000) for 10 min.

### Image acquisition and analysis

Images were acquired using the Opera Phenix High-Content Screening System (Perkin Elmer). Images were acquired with a ×40 objective as confocal *z*-stacks with a *z*-step of 1 μm. Stacks were processed to obtain a maximum intensity projection. A minimum of 12 fields of view were taken for each well. To calculate the nuclear:cytoplasmic ratio of RBPs in single cells, images were analysed using the Columbus Image Analysis System (Perkin Elmer). A DAPI mask defined the nucleus, and based on nuclear properties a trained machine learning feature selected neurons in an automated fashion. For each individual cell, an average nuclear intensity of the RBP of interest was measured. For the cytoplasmic measurement, a 1.5 µm cytoplasmic region was defined around the nucleus within a cytoplasmic mask and an average intensity was measured. An example of this nuclear and cytoplasmic compartments defined by this analysis can be found in Supplementary Fig. 1A. A ratio of the nuclear:cytoplasmic average intensity measurements was calculated per cell. An average of each field was calculated and then averaged across the well.

For the nuclear:neurite ratio, we implemented a semi-automated image analysis pipeline combining Ilastik,^14^ CellProfiler^15^ and ImageJ. Nuclear segmentation was performed using DAPI stained images, in which intensity was scaled in ImageJ from 0 to 500 to allow improved nuclear detection. A randomly selected subset of images was used in Ilastik for generation of a binary nuclear segmentation mask. To define the neurite compartment βIII-tubulin was used to create a neuronal mask as it is a reliable axonal and dendritic marker. To remove the nuclei and cytoplasm from the βIII-tubulin mask the nuclei were expanded by 30 pixels and removed, which ensured only the neurites were included in the analysis. An example of the compartments defined by this analysis can be found in Supplementary Fig. 1B. Intensity measurements of the protein of interest were performed in CellProfiler using the nuclear, and neurite masks. Calculation of intensity values and ratios were performed using custom R scripts.

When examining the nuclear:cytoplasmic or nuclear:neurite ratio, if an increase or decrease in the ratio is detected, it is not apparent which cellular compartment is contributing to the change. To address this for the nuclear:neurite ratio we have utilized the presence of compartment specific markers. The same semi-automated image analysis pipeline was implemented as for the nuclear:neurite ratio as described above, but in addition to the protein of interest, intensity measurements were performed for DAPI and βIII-tubulin and used to calculate the specified ratio.

### Western blot analysis

Protein levels of TDP-43, FUS and SFPQ were assessed in whole cells in control and VCP mutant MNs. Cells were subjected to untreated conditions or treatment of 1 μM of ML240 for 2 h prior to protein extraction. The cells were lysed and proteins were extracted with RIPA disruption. Total protein concentration was quantified using BCA protein assay (Sigma). Equal amounts of protein samples were then loaded onto a gel and separated by SDS PAGE and transferred onto a nitrocellulose membrane. Samples were then blocked with PBS, 0.1% Tween, 5% dry milk powder at RT for 1 h followed by primary antibody incubation overnight at 4°C. The following antibodies were diluted in PBS 5% BSA; TDP-43 (ProteinTech; 12892-1-AP; rabbit; 1:1000), SFPQ (Abcam; 11825; mouse; 1:250), FUS (Santa Cruz; sc-47711; mouse; 1:500), GAPDH (GeneTex; GT239; mouse; 1:10 000). For detection, membranes were incubated with species-specific near infra-red fluorescent antibodies (IRDye, Licor) at RT for 1 h and imaged using an Odyssey Fc Imaging System (Licor).

### Statistical analysis

There are 3 control and 4 *VCP* mutant iPSC lines, with details found in Supplementary Table 1. The number of cells used in each experiment is stated within the figure legends. At a minimum, for each line data is collected from 34 fields of view from 6 wells across 3 independent experimental repeats. Data are plotted as a violin plot, with data plotted as per field or per well and the mean values for each cell line shown. When data are displayed as normalized to untreated control, each raw value has been divided by the average of the untreated control within each experimental repeat. An unpaired two-tailed student’s *t*-test was used when comparing between two individual groups with Gaussian distribution. When Gaussian distribution was not achieved a Mann–Whitney test was used. Statistical analysis was conducted by Prism 8. A *P*-value 0.05 or below was considered to be statistically significant (*P < 0.05, **P < 0.01, ***P < 0.001).

### Data availability

The data that support the findings of this study are available on request from the corresponding author.

## Results

### TDP-43, SFPQ and FUS are mislocalized to neurites in *VCP* mutant MNs

**Figure 1:**
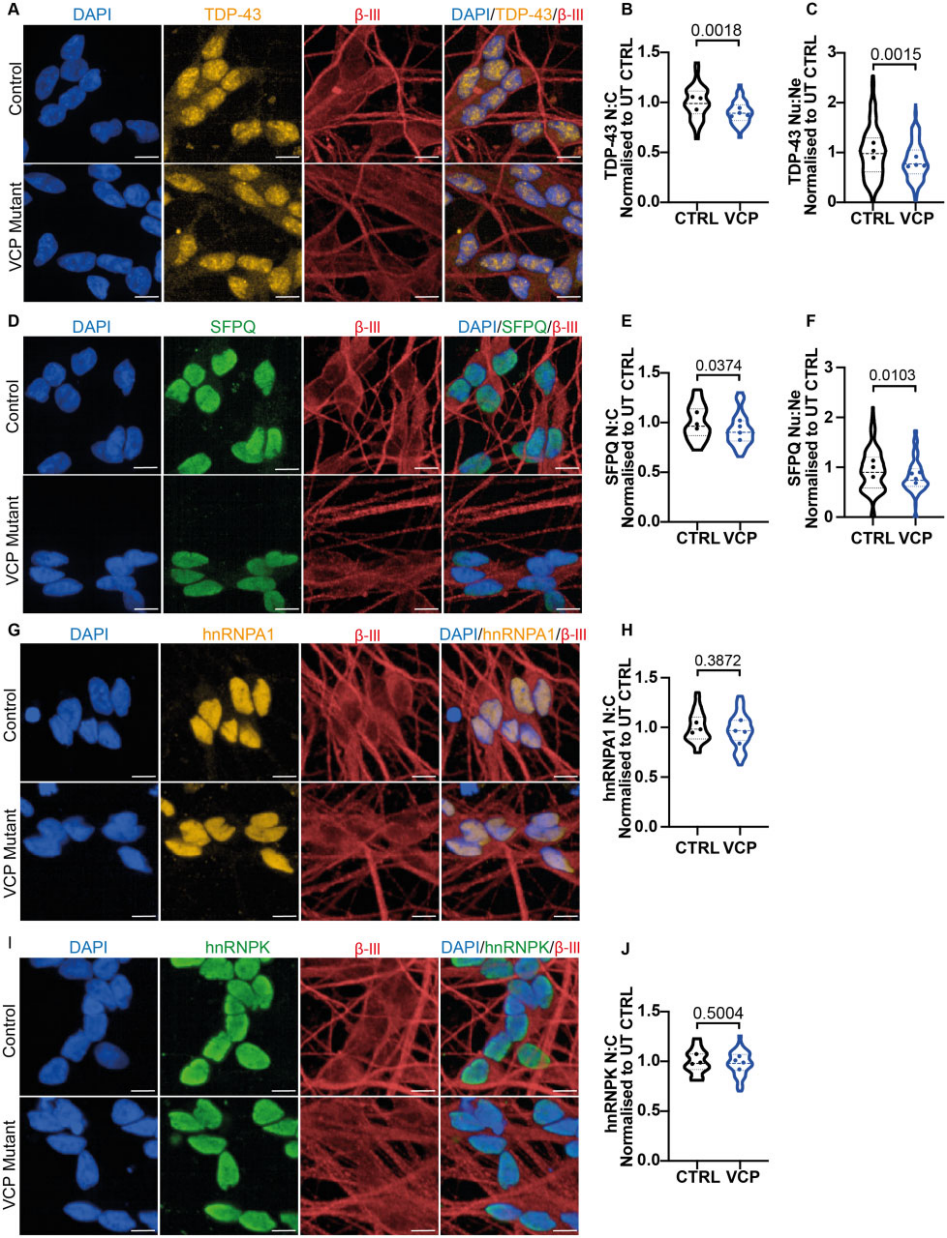
**TDP-43 and SFPQ mislocalization in *VCP* mutant MNs.** (**A**) TDP-43 immunolabelling in control and *VCP* mutant MNs. (**B**) Individual cell analysis of TDP-43 nuclear:cytoplasmic ratio identifies *VCP* mutant MNs display a loss in the nuclear:cytoplasmic ratio (N:C). (**C**) TDP-43 quantification in the neurites of MNs show *VCP* mutant motor neurons have a loss in the nuclear:neurite ratio (Nu:Ne). (**D**) SFPQ immunolabelling in control and *VCP* mutant motor neurons. (**E**) Individual cell analysis of SFPQ nuclear:cytoplasmic ratio shows there is a small but significant loss in *VCP* mutant MNs. (**F**) SFPQ quantification in the neurites of MNs identifies that *VCP* mutant MNs have a decrease in the nuclear:neurite ratio. (**G**) Immunolabelling of hnRNPA1 in control and *VCP* mutant MNs. (**H**) Individual cell quantification of hnRNPA1 shows there is no difference in the nuclear:cytoplasmic ratio in control and *VCP* mutant MNs. (**I**) hnRNPK localization in control and *VCP* mutant MNs. (**J**) Quantification of hnRNPK shows no difference in the nuclear:cytoplasmic ratio in control and *VCP* mutant MNs. Scale bar = 10 μm. Data are collected from 3 control cell lines and 4 *VCP* mutant lines. For graphs B, E, H and J data are shown as a violin plot with data plotted per well from 6 independent experimental repeats and the data points represent the mean value for each cell line. The *P*-value shown is calculated from an unpaired *t*-test. Approximately, the following number of cells were analysed; (**B**) CTRL1:10 000, CTRL2:10 000, CTRL3:14 000, MUT1:13 000, MUT2:15 000, MUT3:14 000, MUT4:11 000, (**E**) CTRL1:9000, CTRL2:10 000, CTRL3:13 000, MUT1:12 000, MUT2:14 000, MUT3:14 000, MUT4:12 000, (**H**) CTRL1:9000, CTRL2:10 000, CTRL3:13 000, MUT1:12 000, MUT2:12 000, MUT3:13 000, MUT4:9000, (**J**) CTRL1:9000, CTRL2:9000, CTRL3:12 000, MUT1:11 000, MUT2:12 000, MUT3:12 000, MUT4:9000. For graphs C and F, data are collected from 3 independent experiments from 3 control and 4 VCP mutant lines, with >5000 neurons analysed for each cell line. Data are shown as a violin plot from fields of view and the data points represent the mean value for each cell line. The *P*-value calculated from a Mann–Whitney test. All data are normalized to the average of the control values in each experimental repeat.

We have utilized our established and robust differentiation of human iPSCs into highly enriched and characterized spinal cord MNs, which were positive for choline acetyltransferase (ChAT), SMI-32 and βIII-tubulin (TUJ1) (Supplementary Fig. 2). Importantly, we have previously functionally validated our enriched MN cultures by (i) demonstrating cytosolic calcium responses to physiological calcium stimuli (glutamate and KCl); (ii) whole-cell patch clamping; (iii) multi-electrode array (MEA) analysis^12^ and (iv) co-culture with iPSC-derived skeletal muscle with demonstration of neuromuscular junction formation.^13^ Using this model, we have previously reported time-resolved pathogenic phenotypes of *VCP*-related ALS, including new hallmarks of ALS such as reduced SFPQ and FUS nuclear-to-cytoplasmic ratio in mutant neural precursors.^2^^,^^3^^,^^12^ Additionally, we have confirmed subcellular TDP-43 and FUS mislocalization phenotypes in terminally differentiated MNs.^12^^,^^16^^,^^17^ However, the majority of our prior studies and those of others have not systematically examined these aforementioned RBPs together, nor addressed the specific site of mislocalization of these RBPs with respect to their presence within neurites. Against this background, we utilized our *VCP* mutant iPSC-derived MNs to comprehensively investigate the subcellular localization of 5 ALS-related RBPs. Single-cell analysis of the nuclear-to-cytoplasmic ratio of >70,000 neurons revealed a decrease in TDP-43 and SFPQ in *VCP* mutant human MNs (Fig. 1A, B, D, E), which builds on our recent report of reduced FUS nuclear-to-cytoplasmic ratio.^16^ Upon further analysis, we detect that TDP-43 and SFPQ additionally have a reduced nuclear-to-neurite ratio and thus are also aberrantly localized within the neurites of *VCP* mutant MNs (Fig. 1C and F). The presence of compartment specific markers (nuclear: DAPI, neurites: βIII-tubulin) enabled us to then examine the nuclear and neurite compartments independently, revealing that the reduced nuclear-to-neurite ratios of these RBPs are driven by both their nuclear loss and neurite gain (Supplementary Fig. 3A–D). To exclude the possibility that that RBPs are generically mislocalized in iPSC models, we next examined the subcellular localization of hnRNPA1 and hnRNPK, which have previously been implicated in ALS.^18^^,^^19^ However, hnRNPA1 and hnRNPK exhibited no detectable change in their nuclear-to-cytoplasmic localization in *VCP* mutant MNs consistent with selective mislocalization of TDP-43, FUS and SFPQ in iPSC-derived *VCP* mutant MNs (Fig. 1G–J).

### Pharmacological inhibition of the VCP D2 ATPase domain does not induce ALS phenotypes in healthy human MNs

**Figure 2:**
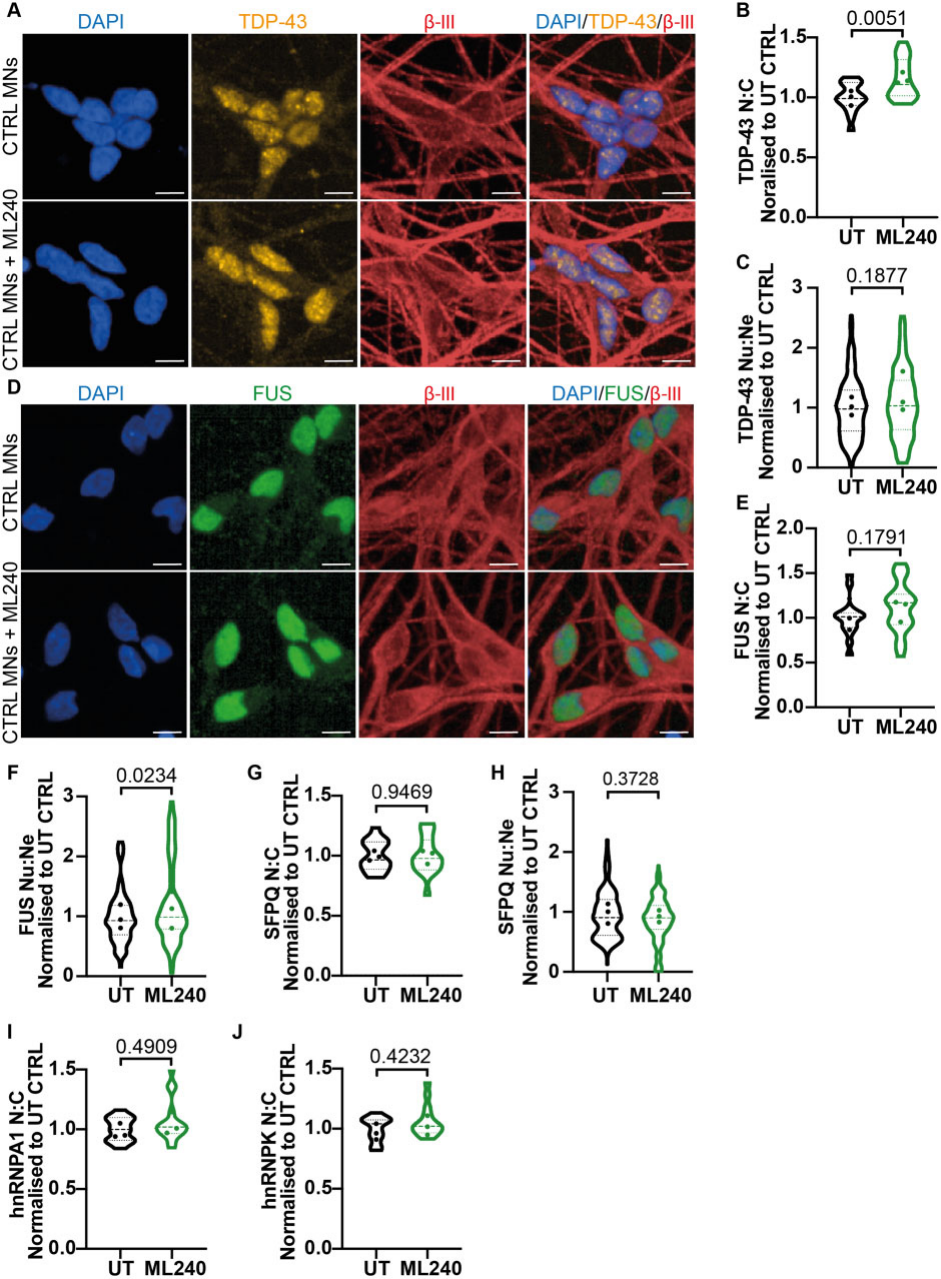
**Pharmacological inhibition of VCP D2 ATPase does not recapitulate ALS RBP mislocalization phenotypes in control MNs.** (**A**) Control MNs treated with 1 μM of ML240 immunolabelled with TDP-43 and βIII-tubulin and a DAPI stain. (**B**) Individual cell quantification of TDP-43 displayed control MNs treated with ML240 results in an increase in the nuclear:cytoplasmic ratio (N:C). (**C**) There was no difference in the nuclear:neurite ratio (Nu:Ne) of TDP-43 upon ML240 treatment. (**D**) Control MNs treated with 1 μM of ML240 immunolabelled with FUS and βIII-tubulin and a DAPI stain. (**E**) Treatment of ML240 to control MNs showed no difference in the nuclear:cytoplasmic localization of FUS. (**F**) A small increase in the nuclear:neurite ratio of FUS upon ML240 treatment was observed. (**G**) There was no difference in the nuclear:cytoplasmic ratio or (**H**) nuclear:neurite ratio of SFPQ upon ML240 treatment. (**I**) There was no difference in the nuclear:cytoplasmic ratio of hnRNPA1 or (**J**) hnRNPK upon ML240 treatment in control MNs. Scale bar = 10 μm. Data are shown as violin plots normalized to control untreated values in each experimental repeat. Data are collected from 3 control lines across 3 independent experimental repeats using approximately the following number of cells in both untreated and treated conditions; CTRL1:3000, CTRL2:6000, CTRL3:6000. Data are shown as a violin plot with data points representing the mean of each cell line. For graphs B, E, G, I, J; data are plotted by well and the *P-*value is calculated from an unpaired t-test, for graphs C, F, H; data are plotted by field of view and the *P-*value is calculated from a Mann–Whitney test.

It has remained controversial in the field if *VCP* disease mutations exert dominant-active or dominant-negative effects. To gain mechanistic insight into the effect of the *VCP* mutations in human MNs, we utilized ML240, a potent and selective inhibitor of the D2 ATPase domain in the VCP protein.^20^ Control MNs were treated with 1 μM of ML240 for 2 h prior to fixation and immunocytochemistry. Interestingly, inhibition of the D2 ATPase *increased* the nuclear-to-cytoplasmic ratio of TDP-43 (Fig. 2A and B). However, no such increase was observed in the nuclear-to-neurite ratio of TDP-43, possibly suggesting a proximal > distal change in protein distribution in this context (Fig. 2C). Interestingly, we found that although FUS nuclear-to-cytoplasmic ratio did not change, a small but statistically significant increase in FUS nuclear-to-neurite ratio was observed (Fig. 2D–F). This suggests that the D2 ATPase domain may have RBP-specific roles in a cell compartment-specific manner. Analysis of the additional aforementioned RBPs; SFPQ, hnRNPA1 and hnRNPK revealed no changes in their nuclear-to-cytoplasmic ratios or nuclear-to-neurite ratio (SFPQ) upon VCP D2 ATPase inhibition (Fig. 2G–I). Together, these data argue against a loss of function of the VCP D2 ATPase domain as a mechanism for the observed RBP mislocalization phenotypes.

### Pharmacological inhibition of the VCP D2 ATPase domain reverses *VCP* mutation-related mislocalization of TDP-43 and FUS in human MNs

**Figure 3:**
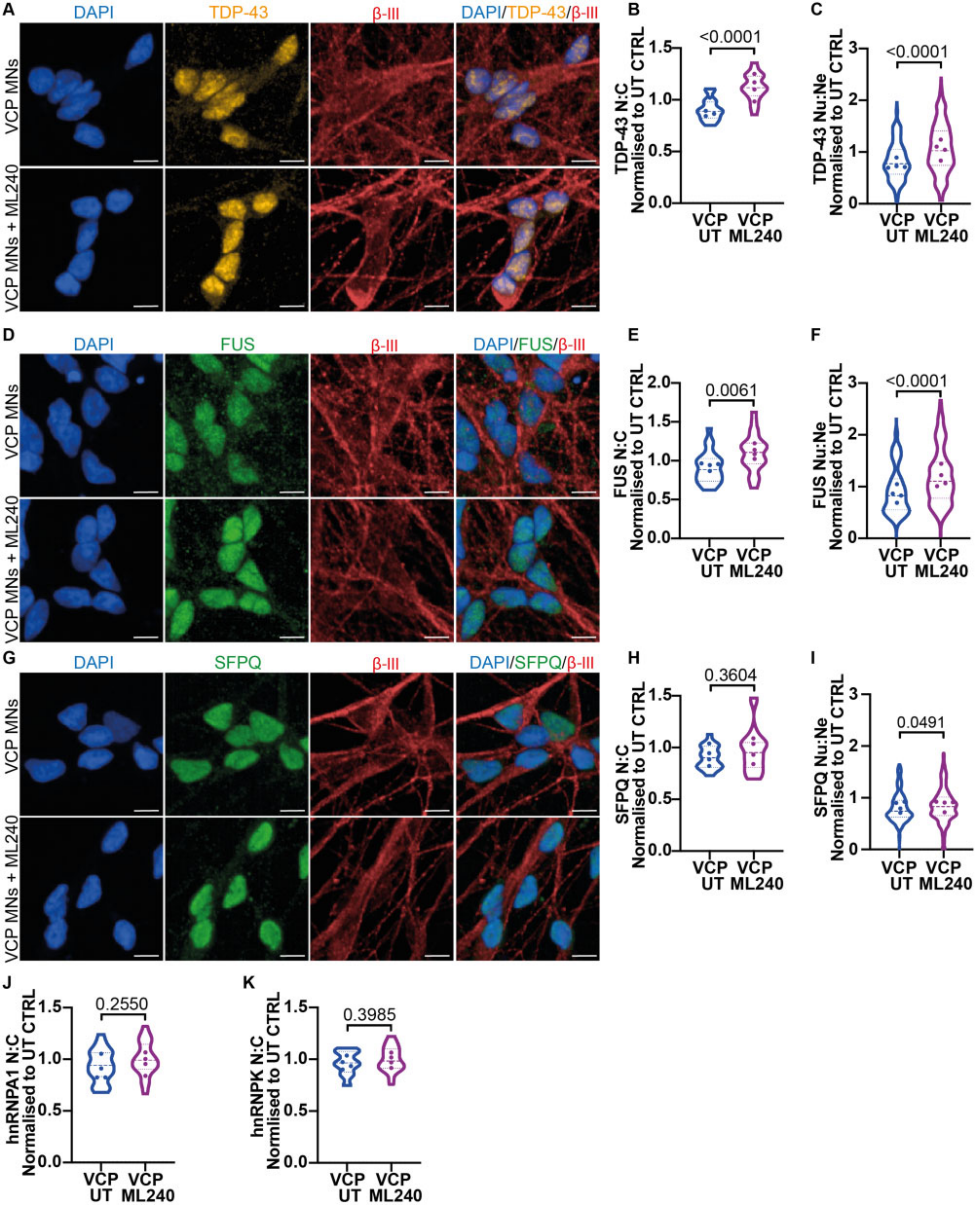
**Inhibition of VCP D2-ATPase domain reverses TDP-43, FUS and SFPQ mislocalization phenotypes in *VCP* mutant MNs.** (**A**) *VCP* mutant MNs treated with 1 μM of ML240 immunolabelled with TDP-43 and βIII-tubulin. (**B**) Cell by cell quantification of the nuclear:cytoplasmic ratio (N:C) shows *VCP* mutant MNs have a loss in the nuclear:cytoplasmic ratio that is increased above control values upon ML240 treatment. (**C**) Quantification of TDP-43 in the neurites shows an increased nuclear:neurite ratio (Nu:Ne) upon ML240 treatment. (**D**) FUS and βIII-tubulin immunolabelling in VCP mutant MNs treated with ML240. (**E**) Quantification of FUS in the nucleus and cytoplasm identify an increase in the nuclear:cytoplasmic ratio upon ML240 treatment in *VCP* mutant MNs. (**F**) Quantification of FUS in the neurites shows an increase of the nuclear:neurite ratio to control values upon ML240 treatment. (**G**) *VCP* mutant MNs treated with ML240 and immunolabelled with SFPQ and βIII-tubulin. (**H**) Treatment of ML240 results in no change in the subcellular distribution of SFPQ when examining the nuclear:cytoplasmic ratio (**I**) but an increase when examining the nuclear:neurite ratio. (**J**) Quantification of hnRNPA1 shows no change in the nuclear:cytoplasmic ratio upon ML240 treatment in VCP mutant MNs. (**K**) Quantification of hnRNPK shows no change in the nuclear:cytoplasmic ratio upon ML240 treatment in VCP mutant MNs. Scale bars = 10 μm. Data are collected from 3 independent experimental repeats from 4 *VCP* mutant ALS lines analysing approximately the following number of cells; MUT1:7000, MUT2:6000, MUT3:7000, MUT4:6000. Data are normalized to control untreated values for each experimental repeat. Data are shown as violin plots with the data points representing the mean for each cell line. Data are plotted by well in graphs B, E, H, J and K and by field of view in graphs C, F and I. Graphs B, E, H and K; *P-*value is calculated from an unpaired *t*-test, for graphs C, F, I and J; *P-*value is calculated from a Mann–Whitney test.

Noting the apparent effect of ML240 on TDP-43 in control MNs, we reasoned that its application to the *VCP* mutant MNs may ameliorate their RBP mislocalization phenotypes. Specifically, we hypothesized that the ALS causing *VCP* mutations (R155C and R191Q) result in a dominant-active effect of the D2 ATPase domain. Application of ML240 in *VCP* mutant MNs indeed robustly reversed the mislocalization of both TDP-43 and FUS when examining both the nuclear-to-cytoplasmic mislocalization and nuclear-to-neurite mislocalization (Fig. 3A–F). For SFPQ, ML240 treatment also significantly reversed the nuclear-to-neurite mislocalization. However, although ML240 treatment resulted in an increase in the nuclear-to-cytoplasmic ratio (Average; UT = 0.90, ML240 = 0.97), this difference did not reach statistical significance for SFPQ (Fig. 3G–I). We further demonstrate that this reversal is due to the relocalization of TDP-43, FUS and SFPQ from the neurites and/or cytoplasm to the nucleus, as overall protein levels by western blot did not change upon ML240 treatment (Supplementary Fig. 4A and B). Notably, hnRNPA1 and hnRNPK exhibited no change in the nuclear-to-cytoplasmic ratio, with ML240 treatment only affecting the localization of RBPs that were significantly mislocalized as a result of the *VCP* mutations (Fig. 3J and K).

## Discussion

ALS is a devastating and incurable neurodegenerative disease. Missense mutations of *VCP* account for 1–2% of familial ALS, but can additionally cause an autosomal dominant disease known as inclusion body myopathy, Paget disease and frontotemporal dementia (IBMPFD). ALS-causing *VCP* mutations recapitulate key hallmarks of sporadic ALS including nuclear-to-cytoplasmic mislocalization of key RBPs including TDP-43, FUS and SFPQ.^2^^,^^3^^,^^8^^,^^16^ However, the mechanism by which *VCP* mutations lead to RBP mislocalization in ALS has remained elusive. In this study, we have used our highly enriched and functionally validated iPSC-derived, patient specific motor neuron model^2^^,^^12^^,^^13^ to systematically investigate the effects of ALS-causing VCP mutations on RBP nucleocytoplasmic localization and the ability of an inhibitor of the VCP D2 ATPase to suppress these VCP-related disease phenotypes. Importantly, this model conveys mutations at pathophysiological levels and does not rely on artificial overexpression, thereby closely approximating ALS MNs.

We first demonstrate that *VCP* mutations (R155C and R191Q) cause selective reduction in the nuclear-to-cytoplasmic ratios of TDP-43, FUS and SFPQ in human MNs, where we observe a normal nucleocytoplasmic distribution of both hnRNPK and hnRNPA1. We further show that TDP-43, FUS and SFPQ are also mislocalized into the neurites of MNs (Fig. 1). Recent research has shown an emerging role for these RBPs in axonal mRNA translation and viability, suggesting that impaired axonal RNA processing could contribute to specific pathophysiology in MNs.^21–23^

The main finding of our work, however, is that *VCP*-mutation related mislocalization of TDP-43, FUS and (in part) SPFQ is reversible through pharmacological inhibition of the D2 ATPase domain of VCP protein, using the potent and selective inhibitor ML240.^11^^,^^20^^,^^24^ This dominant-active *VCP* mutation mechanism has also been shown in relation to other phenotypes.^25^^,^^26^ However, controversy exists in the field with some studies suggesting that VCP mutants function as dominant negatives.^27–29^ These seemingly contrasting studies can be reconciled by raising the hypothesis that inhibiting ATP hydrolysis can reverse downstream effects associated with excess ATP hydrolysis whilst also increasing dominant negative effects on ATPase activation. Furthermore, as VCP has a wide range of intracellular functions, we can hypothesize that *VCP* mutations may result in both dominant active or negative effects, dependent on cofactor binding and subsequent downstream cellular pathways.

The underlying molecular mechanisms of how VCP interacts with TDP-43, FUS and SFPQ is unknown and further studies should investigate this. To date, studies have shown a direct interaction between VCP and RBPs, including TDP-43^30^ and FUS,^31^ but with limited understanding of molecular consequences of these interactions. A recent study in yeast showed a role for Cdc48/VCP in endocytosis-dependent turnover of TDP-43 and FUS.^32^ Taken together with this study, this raises the possibility that an increase in D2 ATPase activity may disrupt VCP-dependent endocytic mechanisms of cytoplasmic proteostasis. Understanding the precise consequences of VCP’s interaction with RBPs will help decipher mechanisms underpinning their mislocalization in disease states.

Our findings that *VCP* disease mutants exhibit increased D2 ATPase activity have potential important therapeutic implications. Indeed, VCP inhibitors have been found to rescue multiple VCP disease phenotypes in drosophila models and patient fibroblasts.^26^ Notably, rescue was multi-faceted including amelioration of mitochondrial phenotypes, p62 and ubiquitin pathology. In the context of our findings, this suggests that VCP D2 ATPase pharmacological inhibitors may be effective across the range of multi-system pathology caused by *VCP* mutations. This extends beyond ALS and IBMPFD into some cases of *VCP*-related Charcot-Marie-Tooth disease and hereditary spastic paraplegia.^33^^,^^34^ However, as studies have shown that VCP inhibitors can perturb cellular homoeostasis in a dose-dependent manner, a therapeutic balance must be investigated and optimized in future studies. However, with VCP inhibitors already in phase I clinical trials for cancer treatment, it is important to recognize the therapeutic potential for these devastating and hitherto incurable diseases.

## Supplementary material

Supplementary material is available at *Brain Communications* online.

## Funding

This work was supported by the Francis Crick Institute which receives its core funding from Cancer Research UK (FC010110), the UK Medical Research Council (FC010110) and the Wellcome Trust (FC010110). R.P. holds an MRC Senior Clinical Fellowship [MR/S006591/1]. A.S. acknowledges the support of the Wellcome Trust [213949/Z/18/Z].

## Competing interests

The authors report no competing interests.

## Supplementary Material

fcab166_Supplementary_Data

## Abbreviations

ALS =: amyotrophic lateral sclerosis
ChAT =: choline acetyltransferase
FUS =: fused in sarcoma/translocated in liposarcoma
hnRNPA1 =: heterogeneous nuclear ribonucleoprotein A1
hnRNPK =: heterogeneous nuclear ribonucleoprotein K
IBMPFD =: Inclusion Body Myopathy, Paget’s disease and Frontotemporal Dementia
iPSCs =: induced pluripotent stem cells
MNs =: motor neurons
RBP =: RNA binding protein
SFPQ =: splicing factor proline and glutamine rich
TDP-43 =: transactive response DNA-binding protein 43
VCP =: valosin-containing protein
