# Supplementary material for: TDP-43 and FUS mislocalization in VCP mutant motor neurons is reversed by pharmacological inhibition of the VCP D2 ATPase domain
Source: Brain Commun. 2021 Aug 6;3(3):fcab166. doi: 10.1093/braincomms/fcab166 (PMC8361416; doi:10.1093/braincomms/fcab166)
Supplement: fcab166_Supplementary_Data [file fcab166_supplementary_data.pdf]

Supplementary Information

IPSC cell line	Mutation	Age of the donor	Age at disease onset	Sex of the donor
CTRL1	None	78	n/a	Male
CTRL2	None	64	n/a	Male
CTRL3	None	unknown	n/a	Female
MUT1	R191Q	42	36	Male
MUT2	R191Q	42	36	Male
MUT3	R155C	43	40	Female
MUT4	R155C	43	40	Female

Supplementary Table 1. *Details of the iPSC cell lines used in the study.*

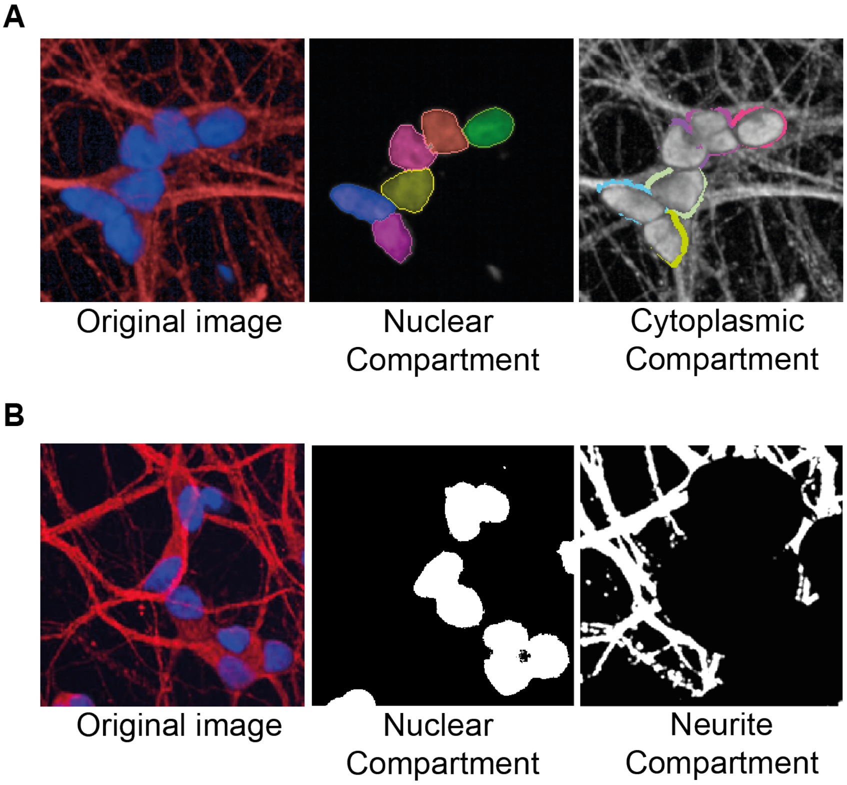

Supplementary Figure 1. Example images of the neuronal segmentation used in the image analysis. A) Examples of nuclear and cytoplasmic compartments used in the nuclear:cytoplasmic ratio analysis. The nuclear:cytoplasmic ratio is calculated per cell. B) Example of the nuclear and neurite compartments used in the nuclear:neurite ratio analysis. The nuclear:neurite ratio is calculated per field of view.

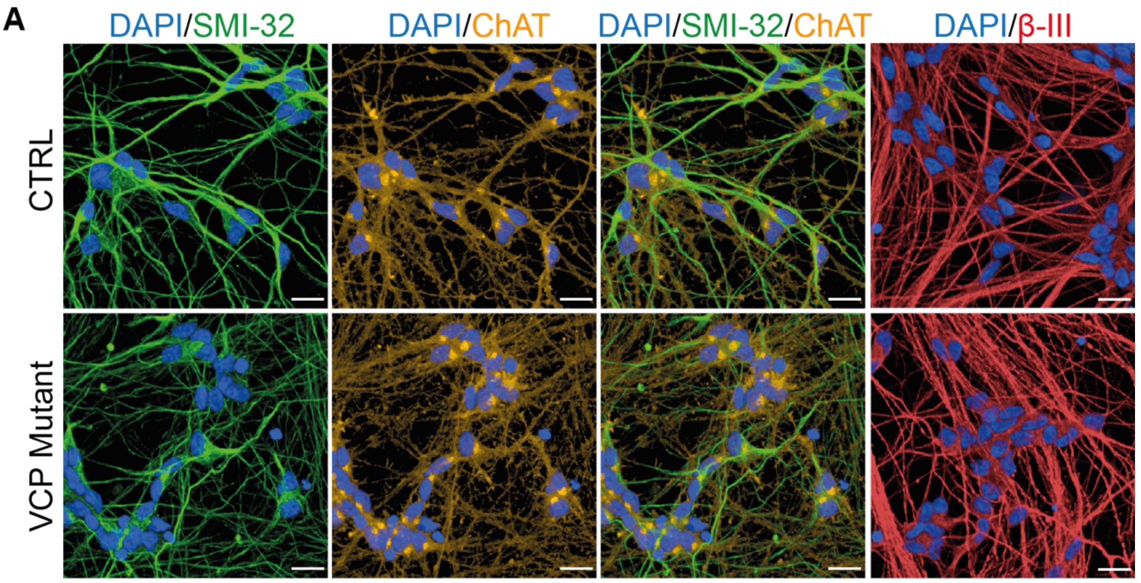

Supplementary Figure 2. Motor neuron characterisation. Representative images of control and VCP mutant iPSC-derived motor neurons, immunolabeled with motor neuron specific markers SMI-32 and ChAT and neuronal marker βIII-tubulin. Scale bar = 20μm.

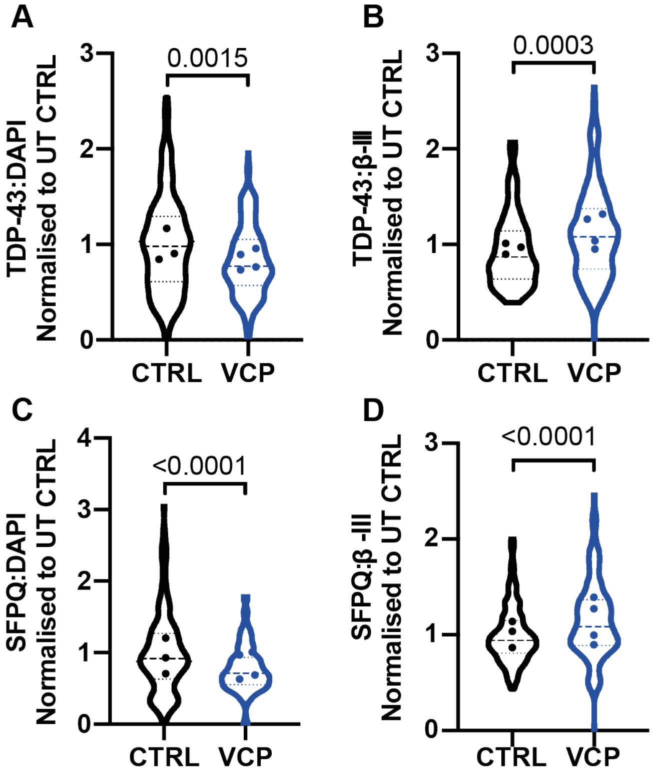

Supplementary Figure 3. Compartmental analysis of TDP-43 and FUS in VCP mutant motor neurons. A) Nuclear compartmental analysis shows VCP mutant motor neurons have a loss of TDP-43 in the nucleus when compared to DAPI. B) Neurite compartmental analysis shows VCP mutant motor neurons have a gain of TDP-43 in the neuronal process when compared to neuronal marker βIII-tubulin. C) Compartmental analysis shows a loss of SFPQ protein in the nucleus in VCP mutant motor neurons. D) Compartmental analysis shows a gain of SFPQ in the neurites of VCP mutant motor neurons. Data is shown as violin plots

normalised to control untreated values in each experimental repeat. Data is collected from 3 control lines and 4 VCP mutant lines from 6 wells across 3 independent experimental repeats. Data is plotted per field of view and the data points represent the mean of each cell line. The *p* value is calculated from a Mann-Whitney test.

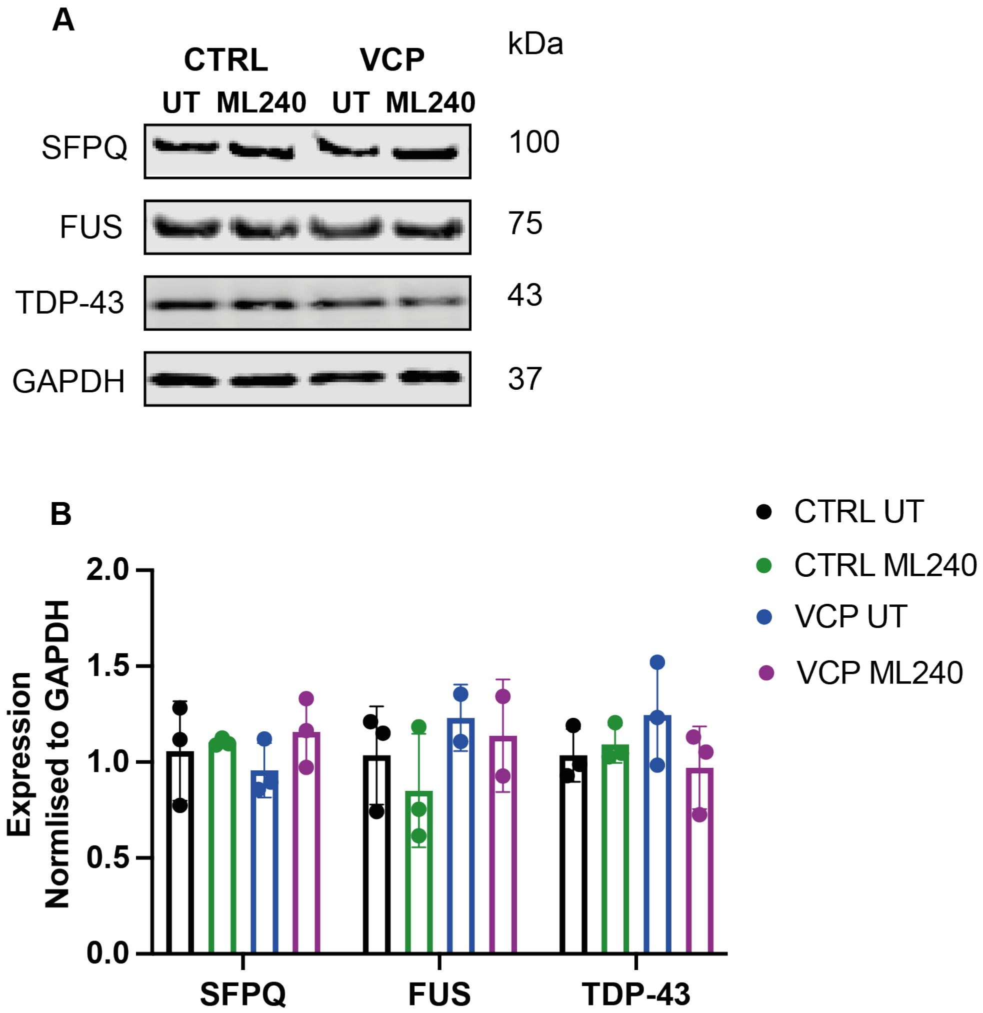

Supplementary Figure 4. Western blot analysis shows TDP-43, FUS and SFPQ protein levels do not change upon inhibition of VCP D2 ATPase domain. A) Representative immunoblot of SPFQ, FUS and TDP-43 in control and VCP mutant MNs untreated and treated with ML240 1μM. B) Quantification of SPFQ, FUS and TDP-43 from 3 control and 3 VCP mutant lines normalised to GAPDH showed ML240 treatment does not change overall protein levels.

Supplementary Methods

Motor neuron differentiation

IPSC were plated to 100% confluency and then differentiated to the neuroepithelium in medium consisting of DMEM/F12 Glutamax, Neurobasal, L-Glutamine, N2 supplement, nonessential amino acids, B27 supplement, β-mercaptoethanol (all from Life Technologies) and insulin (Sigma). The cells underwent a sequential treatment with small molecules, with day 0-7: 1μM Dorsomorphin (Millipore), 2μM SB431542 (Tocris Bioscience), and 3.3μM CHIR99021 (Miltenyi Biotec), day 7-14: 0.5μM retinoic acid (Sigma) and 1μM Purmorphamine (Sigma), day 14-18: 0.1μM Purmorphamine. Following 18 days of neural conversion and patterning, neural precursors were terminally differentiated in 0.1μM Compound E (Enzo Life Sciences). Throughout the neuroepithelial layer was enzymatically dissociated using dispase (GIBCO, 1 mg ml-1). The neural precursors were dissociated with Accutase (Life Technologies) for final plating onto a 96 well plate (Falcon) coated with polyethylenimine (PEI) (2.2mg/ml in 0.1M of sodium borate (Sigma) and Geltrex. Following 6 days of terminal differentiation, cells were fixed in 4% paraformaldehyde for immunolabeling.
